# Microbial Musings – September 2021

**DOI:** 10.1099/mic.0.001115

**Published:** 2021-10-21

**Authors:** Gavin H. Thomas

**Affiliations:** ^1^​ Department of Biology, University of York, YO10 5YW, UK

This is a month about communities, both of people and of microbes, and how microbiology can play important roles in science communication and education. Our hashtag #publishingforthecommunity emphasises how publishing in Microbiology Society journals, and in fact most learned society journals, ensures that the profits of publishing are invested directly back in promoting the subjects closest to our hearts. First up this month is the highly readable Peter Wildy Prize Lecture which was delivered by Graham Hatfull (@GHatfull) from the University of Pittsburgh, USA, at our annual conference back in the Spring [1]. Graham has been a leading proponent of an interesting initiative called SEA-PHAGES, to engage high school pupils into science by offering simple, but highly rewarding, research projects that can be run at scale and across multiple institutions. SEA-PHAGES now runs across over 150 schools with over 5000 students participating annually. The microbiology behind this is the discovery of new bacteriophage, with the students screening diverse soil or compost samples in their hunt. Each student does a simple phage extraction, mixing this with a bacterial culture and plating, followed by recovery of resulting phage after plaquing. This can then be purified and the phage DNA sequenced, annotated and compared to other phage. Due to the enormous diversity of phage in the environment many of the students get to name their own discovery, which must be a pretty cool experience for them and as Hatfull’s article title suggests ‘Who wouldn’t want to discover a new virus?’

This interest in phage diversity and function sits at the heart of Hatfull’s own research and he also outlined a very important application of his vast collections of phage [2]. This was in response to a direct request from clinicians led by Dr. Helen Spencer (@DrHelenSpencer) at Great Ormond Street Hospital in London, UK, for new ways to help two young cystic fibrosis (CF) patients who had contracted *Mycobacterium abscessus *, a drug-resistant opportunistic pathogen, which has very poor outcomes for CF patients [3]. In fact I was just reading some further great work from Julian Parkhill and colleagues at the University of Cambridge, UK, extending their analysis of the global distribution of this microbe and their findings that rather than independent environmental origins of strains that infect CF patients they appear to originate from a small number of circulating clones, which they now propose originated in non-CF patients and were then spread through the CF community [4]. Anyway, it is a highly dangerous pathogen for CF patients and Hatfull and colleagues used an existing library of phage in their collections, that were active against the related bacterium *Mycobacterium smegmatis* [2]. For one of the patients no active phage were found and sadly the patient passed away shortly afterwards, however, for the other a cocktail of phage were found and used successfully to stabilise the patient. The longer terms application of these phage needs more research but holds much promise and this was an exciting application of phage that could have even been discovered by a community route. An inspiring example of how well-thought-out science education projects can have real impact. Hatfull concludes by thinking about how project attributes from SEA-PHAGES could be applied to other ‘science in the community’ projects [5] and challenges to us to find other examples of programmes that could be developed in similar ways.

Our second paper this month sticks with microbes isolated from soil and community-led contributions [6]. In this new paper from Microbiology Society Fleming Prize winner Nicola Stanley-Wall (@bacteriacities) from Dundee University, UK, with long-term collaborator Cait MacPhee (@sciorama) from Edinburgh University, UK, the authors build on a citizen science project in Dundee where locals collected soil samples and brought them into the University to isolate bacteria contained within them. As well as giving the participants a ‘diversity’ plate to show them the manifold types of different microbes present in their soil, they used the same materials to isolate specifically *Bacillus subtilis* strains which they added to their collection of biofilm-forming isolates. Using this collection of environmental isolates the authors, led by Margarita Kalamara, assessed their diversity by measuring a classical biofilm phenotype of this species, namely the ability to form a hydrophobic surface on a colony biofilm [7]. Surprisingly, while the genome sequences of the strains showed that they all contained the *bslA* gene that encodes the BslA protein required for this phenotype [8, 9] and that the protein itself could be detected in the cells by Western blotting, only a small number of the twenty or so strains displayed the hydrophobic biofilm phenotype. In the few strains that did have the hydrophobic phenotype, this was dependent on the presence of *bslA* as it was lost when the genes was disrupted. Given that all the strains make BslA, they thought that this unexpected phenotypic diversity might be due to BslA function being ‘masked’ by another biofilm component, namely the hydrophilic poly γ-glutamic acid (γ-PGA) polymer, but *pgsB* mutants lacking this cell surface feature do not alter the hydrophobic phenotype. Hence, it remains to be discovered what other factors lead to the observed phenotypic heterogeneity and the authors speculate that other cell surface structures such as a known exopolysaccharide [10] and other factors that alter the monomeric/dimeric state of BslA could be involved. The data do support the observation that the biofilm phenotype is complex and varies significantly in the environment, which is supported by some work from Ákos Kovác’s (@EvolvedBiofilm) lab at DTU, Denmark, showing that experimental evolution of a isogenic biofilm of *B. subtilis* can readily differentiate into different morphotypes, including those that are now hydrophilic [11].

Our third paper again sticks with soil bacteria and those from the genus Actinobacteria, being the second Actinobase-led community review of actinomycete research [12]. Having commissioned a group of early career researchers (ECRs) to write the first of these in 2020 [13], I am delighted this has continued in the same model for 2021 with a host of new authors coming together in what is probably one of our most international papers of all time! With an authorship of Twitter savvy ECRs comprising of Agustina Undabarrena (@AgusUndabarrena), Camila Pereira (@CamilaFiori2), Worarat Kruasuwan (@birdkruasuwan), Jonathan Parra (@JonathanParraV), Nelly Sélem-Mojica (@nselem35), Kristiina Vind (@KristiinaVind) and Jana Schniete (@Janitensen), from Chile, Brazil, Mexico, Thailand, the Netherlands and the UK, the authors together review a series of new papers under the general themes of technology and methodology, specialised metabolites, development and regulation and ecology and host interactions. This is a great effort and a very useful way to keep abreast of some important new papers in the field, including new bioinformatics tools for natural product synthesis and metabolomics analysis, such as Big-SLICE [14], BiG-FAM [15] and Qemistree [16] and well as a host of other experimental papers on fundamental and applied discoveries in these bacteria. A must read for any ‘Actino’ researchers out there.

For the final paper of this month, we switch to a paper studying the dynamics of gene transfer in bacterial communities [17]. Almost all molecular biologists will have grown cultures of *Escherichia coli* in the laboratory containing a plasmid. In almost every case both the bacterium will have been grown in pure, axenic culture and only a single plasmid will have been present in the bacterium. However, how does this align with real environments and how could these differences tell us more about natural plasmid carriage and spread? This is what Anastasia Kottaro (@akottara) from the group of *Microbiology* Senior Editor Mike Brockhurst (@BrockhurstLab) at the University of Manchester, UK, set out to investigate, with collaborators Laura Carrilero and Ellie Harrison (@ellieevolves) from the University of Sheffield, UK, and *Microbiology* Editor Jamie Hall (@jpjhall) from the University of Liverpool, UK. Their study was designed to examine a concept first developed in the study of parasite transmission [18]. This idea, known as the ‘dilution effect’, is a phenomenon whereby the infection rate of a focal host species by an infectious agent is reduced in the presence of other species that are less proficient at carriage of that agent. Here they were considering the plasmid as the infectious agent and created an experimental system with two different conjugative plasmids and five species of *Pseudomonas *, including the focal species *P. fluorescens* SBW25. They found that the dilution effect was occurring in this setup with the rates of dual plasmid carriage in the focal species reduced in the presence of the community. They also found evidence for lower rates of transfer of conjugative plasmids when there were two plasmids in the donor strain rather than a single one, supporting previous ideas that there could be plasmid-plasmid interactions that modulate conjugation rate [19]. Overall, the study is important and makes us think harder about how we study plasmids in bacterial systems due to their natural occurrence in microbial communities with multiples plasmids and multiple species. Here, the authors have clearly demonstrated that there are influences from both these factors in how an individual plasmid can spread, which could also have important applications if it could be used to limit the spread of plasmids encoding multiple antibiotic resistance genes, for example. Next month it looks like we have some cracking papers on bacterial virulence, including our first paper submitted and accepted through our new 'rapid-review' process.

Gavin H. Thomas

Department of Biology, University of York, UK, YO10 5YW.
